# Melatonin modulates TLR4/MyD88/NF-κB signaling pathway to ameliorate cognitive impairment in sleep-deprived rats

**DOI:** 10.3389/fphar.2024.1430599

**Published:** 2024-07-19

**Authors:** Chao Yin, Meiya Zhang, Li Cheng, Li Ding, Qing Lv, Zixuan Huang, Jiaqi Zhou, Jianmei Chen, Ping Wang, Shunbo Zhang, Qiuyun You

**Affiliations:** ^1^ School of Pharmacy, Hubei University of Chinese Medicine, Wuhan, China; ^2^ Engineering Research Center of TCM Protection Technology and New Product Development for the Elderly Brain Health, Ministry of Education, Hubei University of Chinese Medicine, Wuhan, China; ^3^ Hubei Shizhen Laboratory, Hubei University of Chinese Medicine, Wuhan, China

**Keywords:** melatonin, sleep deprivation, cognitive impairmen, TLR4/MYD88/NF-κB signaling pathway, neuroinflammation

## Abstract

Sleep deprivation (SD) is commonplace in today’s fast-paced society. SD is a severe public health problem globally since it may cause cognitive decline and even neurodegenerative disorders like Alzheimer’s disease. Melatonin (MT) is a natural chemical secreted by the pineal gland with neuroprotective effects. The purpose of this study was to investigate the protective effect and mechanism of MT on chronic sleep deprivation-induced cognitive impairment. A 3-week modified multi-platform method was used to create the SD rat model. The Morris water maze test (MWM), Tissue staining (including Hematoxylin and Eosin (H & E) staining, Nissl staining, and immunofluorescence), Western blot, Enzyme-linked immunosorbent assay (ELISA), and Quantitative real-time polymerase chain reaction (qPCR) were used to investigate the protective effect and mechanism of MT in ameliorating cognitive impairment in SD rats. The results showed that MT (50 and 100 mg/kg) significantly improved cognitive function in rats, as evidenced by a shortening of escape latency and increased time of crossing the platform and time spent in the quadrant. Additionally, MT therapy alleviated hippocampus neurodegeneration and neuronal loss while lowering levels of pathogenic factors (LPS) and inflammatory indicators (IL-1β, IL-6, TNF-α, iNOS, and COX2). Furthermore, MT treatment reversed the high expression of Aβ42 and Iba1 as well as the low expression of ZO-1 and occludin, and inhibited the SD-induced TLR4/MyD88/NF-κB signaling pathway. In summary, MT ameliorated spatial recognition and learning memory dysfunction in SD rats by reducing neuroinflammation and increasing neuroprotection while inhibiting the TLR4/MyD88/NF-κB signaling pathway. Our study supports the use of MT as an alternate treatment for SD with cognitive impairment.

## Introduction

A healthy sleep is essential for the rejuvenation of the animal’s body and mind, yet sleep deprivation (SD) or sleep disruption is commonplace in contemporary times due to the accelerated pace of life (Chang et al., 2021; Wu et al., 2022). Sleep disorder syndrome known as SD occurs when a patient has trouble falling or staying asleep, and their sleep experiences worse quality, which ultimately causes problems such as cognitive decline (Chang et al., 2009; Wang Z. et al., 2021). The World Health Organization (WHO) reported that 27% of people worldwide suffered from sleep disorders, with a prevalence of 27%, and the incidence of adult insomnia in China was even as high as 38.2% (Chang et al., 2021). Sleep disorders are not only directly related to cardiovascular diseases such as obesity and hypertension (Hua et al., 2021), but also lead to cognitive decline and even induce neurodegenerative diseases such as Alzheimer’s disease (Teichman et al., 2020; Li et al., 2021), which seriously affects the patient’s quality of life. Improving sleep or intervening in the impairments caused by SD may be the key to preserving cognitive function. In summary, SD has become a serious public health threat worldwide.

The mechanisms of SD-induced cognitive impairment are complex, with neuroinflammation being the most prominent (He et al., 2021; Serna-Rodriguez et al., 2022). Microglia, which are immune cells resident in the brain, are activated under SD stress, resulting in the upregulation of pro-inflammatory mediators, which are critical for the onset and progression of neuroinflammation and cognitive dysfunction in the brain (Qu et al., 2021). Therefore, correcting the abnormal activation of microglia is critical for the therapy of cognitive deficits caused by SD (Qiu et al., 2021). It has been reported that the sleep-wake cycle regulates brain Aβ levels, with increased synthesis during waking hours and higher clearance during sleep (Shokri-Kojori et al., 2018). SD triggers the accumulation and deposition of Aβ in the brain, which causes neuroinflammation and cognitive impairment (Wang and Holtzman, 2020). Cumulative studies have shown that Aβ-induced neuroinflammation is inextricably linked to the activation of the NF-κB signaling pathway mediated by TLR4 (Zhao et al., 2019; Wang Z. et al., 2021). Toll-like receptor (TLR), the body’s first line of immune defense against invasion, is also expressed in microglia as a type I transmembrane protein composed of extracellular and cytoplasmic structural domains (Yang J. et al., 2020). Lipopolysaccharide (LPS) can act on TLR4 receptors in microglia to trigger the innate immune system, modulate the transcriptional regulation of NF-κB, and promote the production and release of pro-inflammatory cytokines (Muhammad et al., 2019; Qu et al., 2021), resulting in hippocampal neurological damage and loss, as well as cognitive impairment in animal models. Increasingly, inhibition of the TLR4/NF-κB signaling pathway has been found to ameliorate neuroinflammation and cognitive dysfunction (Yang et al., 2022) and to be involved in regulating the survival and differentiation of neuroglia and hippocampal neuronal cells.

There is a consensus that SD impairs brain function, but it is noteworthy that SD is particularly damaging to the hippocampus (Li et al., 2022). The hippocampus is a major brain area that governs higher intellectual activities such as learning and memory (Chang et al., 2021), and is critical for the maintenance of emotion, cognition, and memory. The hippocampus includes key regions such as CA1, CA3, CA4, and DG, which play unique roles in both memory formation and consolidation. SD-induced oxidative stress disrupts the redox system and also destroys the hippocampus ultrastructure, leading to vertebral neuron loss and cognitive impairment (Li et al., 2022). The immune system is also affected when the organism is stressed by SD, and the hippocampus is rich in microglia and inflammatory factor receptors capable of responding to inflammation (Qiu et al., 2021). There is evidence that SD lowers hippocampal neuroplasticity and neurogenesis, as well as the hippocampus’ ability to produce long-term potentiation (LTP) (Qiu et al., 2021). Prolonged chronic sleep deprivation inhibits hippocampal cell proliferation and reduces neuronal survival, leading to impaired and deteriorated neurobehavior such as cognition and memory (Li et al., 2022). Clinical studies have revealed that SD-induced inflammatory events caused sustained negative effects on the central nervous system, and hippocampal inflammation greatly impaired cognitive behavior in individuals (Sun et al., 2022). Therefore, ameliorating chronic inflammation in the brain and reducing neuronal damage and loss may be an effective strategy to protect cognitive function in SD patients (Qu et al., 2021).

Melatonin (MT), a natural chemical widely found in vertebrates and secreted mainly by the pineal gland (Alzoubi et al., 2016), tightly regulates circadian rhythms including the sleep-wake cycle (Wang X. et al., 2021), and exerts neuroprotective properties in SD models (Li et al., 2020). In the past few years, MT has been noticed for its enhanced neuroplasticity (Gao et al., 2021), antioxidant (Wang X. et al., 2021), anti-inflammatory (Wang et al., 2023) and anti-obesity (Yin et al., 2020) activities. Although the disruption of neurological function by SD has been reported (Wang et al., 2023), the potential mechanism of MT in protecting or rescuing neuronal function after SD remains to be explored.

Therefore, the purpose of this study was to investigate the protective effect and mechanism of MT on chronic sleep deprivation-induced cognitive impairment, which would be beneficial for the development of MT as an alternative therapy for SD with cognitive impairment.

## Materials and methods

### Reagents and apparatus

**FIGURE 1:**
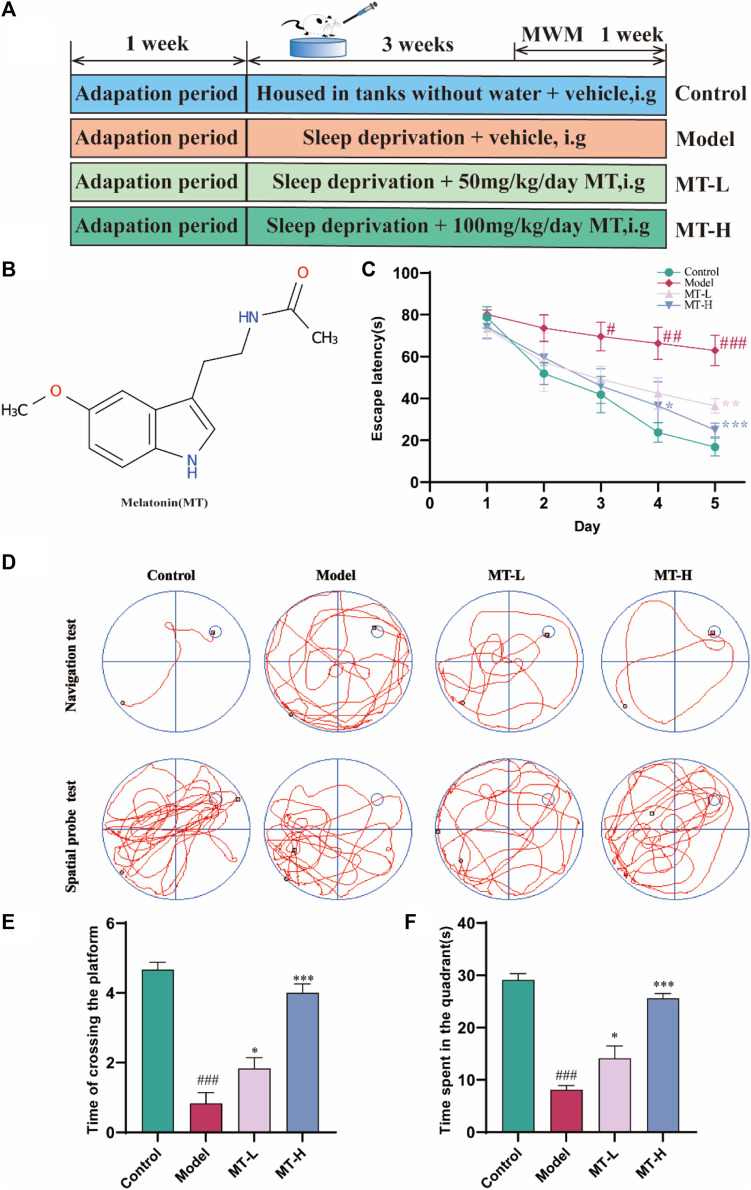
MT ameliorated cognitive impairment in sleep-deprived rats. **(A)** Experimental design and procedure. **(B)** Molecular structure of MT. **(C)** Escape latency. **(D)** Swimming trajectories of rats in Navigation test and Spatial probe test. **(E)** Time of crossing the platform. **(F)** Time spent in the quadrant. The data are expressed as the means ± SEM. ^#^ *p* < 0.05, ^###^ *p* < 0.001 vs. Control group; ^*^ *p* < 0.05, ^**^ *p* < 0.01 vs. Model group.

Shanghai Yuanye Bio-Technology Co., Ltd. (Shanghai, China) provided Melatonin (CAS: 73–31–4, purity > 98%) (Figure 1B). The Carboxymethyl Cellulose Sodium (CMC-Na) was purchased from Sigma-Aldrich (St Louis, MO, United States). The sleep deprivation device was created by the Institute of Gerontology, HBUCM. The Institute of Materia Medica at Chinese Academy of Medical Sciences designed the Morris water maze (MWM) experimental equipment (DMS-2). Noldus Information Technology Co., Ltd. provided the rat trajectory tracking system.

### Animals and treatments

Healthy Sprague-Dawley rats (Male, 200 ± 20 g) were obtained from the Liaoning Changsheng Biotechnology Co. Ltd. (SCXK (Liao) 2020–0001). The Animal Experiment Ethics Committee of HBUCM reviewed and approved the experiment on 1 August 2022 (ethical approval number: HUCMS210726227631713255). Our work followed the rules for the use of animals and humans in neuroscience research. All rats were kept in a 12 h light/dark cycle and had free access to water and feed. The relative air moisture and room temperature were 55% ± 15% and 24°C ± 2°C, respectively. After 7 days of adaptive feeding, thirty-two rats were randomly divided into four groups (n = 8): control group, model group, as well as low- (50 mg/kg, MT-L), and high-dose (100 mg/kg, MT-H) MT group. Next, rats in all groups except the control group were subjected to sleep deprivation for 21 days. Meanwhile, animals in the MT group (50 and 100 mg/kg) were intragastrically administered with the corresponding different concentrations of drugs daily, the two melatonin doses previously proved to be effective in animals (Alzoubi et al., 2016; Wang X. et al., 2021). Animals in the contorl and model groups received equivalent volumes of 0.5% CMC-Na aqueous solution to ensure isocaloric intake. Then the cognitive abilities of these rats were tested by the Morris water maze test (As shown in Figure 1A). After the behavioral tests were completed, all animals were anesthetized by intraperitoneal injection of pentobarbital sodium (40 mg/kg) and killed. All rats’ brain tissues were immediately dissected on an ice plate. Three randomly selected brain tissues from each group were immersed in 4% paraformaldehyde for section staining, while the remaining samples’ hippocampus tissues were separated and immediately frozen in liquid nitrogen for 30 min and finally kept at −80°C.

### Induction of the chronic SD model

The SD model was established by the modified multi-platform method as previously described (Li et al., 2022; Wang et al., 2023). Briefly, several interconnected stainless steel cylinders were placed in the water tank, and these platforms were raised above the water surface by about 1 cm. Therefore, the animals fell asleep causing their noses or heads to touch the water, or even their entire bodies to fall into the water, where they were woken by the watery environment. SD lasted for 21 days, 18 h per day. The control group was kept in tanks without water.

### Morris water maze test (MWM)

As previously stated, spatial learning/memory was investigated using the MWM test (Yin et al., 2024). All rats had their swimming paths, escape latencies, and platform-crossing timings electronically recorded. Reduced deadlines for achieving exam objectives demonstrated mental acuity.

### Hematoxylin and eosin (H and E) staining

Following the descriptions in earlier research (He et al., 2021; Yin et al., 2024), the brain tissues were immersed in 4% paraformaldehyde for 24 h, then paraffin-embedded and cut into 4-μm-thick slices using a microtome. Sections were deparaffinized with xylene and subsequently dehydrated in a gradient of ethanol (70%, 80%, 90%, and 100%, respectively). Afterwards, they were stained sequentially with hematoxylin solution and eosin solution. After sealing the slices, morphological changes in the hippocampal DG region were examined by light microscopy.

### Nissl staining

Paraffin-embedded slices were deparaffinized with xylene for 5 min before being dehydrated in graded ethanol at decreasing concentrations (100%, 95%, 80%, and 70%, respectively). After dehydration, the slices were stained with Nissen for 30 min at 37°C before being rinsed with distilled water. The paraffin slices were then dehydrated in a succession of increasing concentrations of graded ethanol (70%, 80%, 95%, and 100%, respectively), made transparent using xylene, and lastly coated with neutral gum. Images were captured and examined with imaging equipment (BX50, Olympus, Japan). The number of neurons in the hippocampus was analyzed using Image-Pro Plus 7.0 software.

### Immunofluorescence

Immunofluorescence was carried out as previously described (Yin et al., 2024). In brief, tissue slices were treated with primary antibodies (Aβ42, ABclonal, A17911; Iba1, ptgcn, 26177-1-ap, respectively) for 1 h at room temperature, followed by three PBS washes. The secondary antibody was applied to the slices, which were incubated for 1 h at room temperature in a humidified box before being rinsed three times with PBS. A drop of Gelvatol was placed in the center of the coverslip, which was then placed on the slide, covered with aluminum foil, and left in the dark for 30 min to set. The results were seen under a microscope or kept in a slide box at 4°C. Image-Pro Plus 7.0 software was used to stoichiometrically analyze Aβ42 or Iba1 fluorescence intensity in the hippocampal DG region.

### Enzyme-linked immunosorbent assay (ELISA)

The ELISA kit was used to determine the level of rat hippocampus LPS endotoxin. Ruixin Biotechnology Company (Fujian, China) provided the kit (Rat. NO. RX303098R). The whole examination was carried out according to the instructions.

### Western blot

In short, RIPA buffer (P0013B, Bain-Marie Biotechnology, Shanghai, China) was used to lyse and extract the proteins from the tissues. Following reduced SDS-PAGE electrophoresis, 30 μg proteins were separated and placed onto 0.45 μm PVDF membrane (IPVH00010, Millipore). The membrane was then incubated with the primary antibody for an overnight period at 4°C. The primary antibodys used in this experiment are as follows: iNOS(1:2000, BS-0162R, bosterbio), IL-1β(1:2000, BS-0812R, biosscn), TNF-α(1:2000, BA0131, bosterbio), IL-6 (1:2000, BA4339, bosterbio), COX2 (1:5000, ab179800, abcam), IBA1(1:1000, 26177-1-ap, ptgcn), Aβ42 (1:2000, A17911, Abclonal), Occludin (1:5000, 21773-1-Ap, ptgcn), ZO-1 (1:5000, 21773-1-Ap, ptgcn), TLR4 (1:3000, 66350-1-IG, ptgcn), NF-κB p65 (1:2000, bs-20159r, biosscn), IKB-α(1:5000, 10268-1-AP, ptgcn), MyD88 (1:2000, PB9148, bosterbio), p-NF-κB p65 (1:2000, AF 2006, Affinity), p-IKB-α(1:1000, bsm-52169R, biosscn). The HRP-linked secondary antibody (1:5000, SA00001-1; ptgcn) was incubated on the membrane for 2 h at room temperature the next day. The blots were observed by the LAS4000 chemiluminescence system (Fujifilm, Tokyo, Japan). Image-Pro Plus 7.0 software was used to analyze each sample’s band intensity.

### Quantitative real-time polymerase chain reaction (qPCR)

The RNA Isolation Kit (RNAisoPlus, Takara, Japan) was utilized to extract total mRNA. The TransScript^®^ Uni All-in-One First-Strand cDNA Synthesis SuperMix for qPCR (One-Step gDNA Removal) (TransGen Biotech Co.) was used for the reverse transcription (RT) reaction. The PerfectStart^®^ Green qPCR SuperMix (+Dye II) (TransGen Biotech Co.) was employed for the qPCR reaction. The entire PCR experiment was performed completely according to the manufacturer’s instructions. Table 1 displays the primers used in this experiment. The relative expression of mRNA was normalized using β-actin. The 2^−ΔΔCt^ method was then used to analyze the relative expression of mRNA.

**TABLE 1:** Primer sequences for qPCR.

Gene	Forward primer	Reverse primer
TLR4	CCG​CTC​TGG​CAT​CAT​CTT​CA	TGG​GTT​TTA​GGC​GCA​GAG​TT
Occludin	AAT​TTC​GGT​GAG​CGG​TTC​TTC​T	TCA​AGG​CTC​CCA​AGA​CAA​GTG
ZO-1	ATC​CCA​CAA​GGA​GCC​ATT​CC	TCA​CAG​TGT​GGC​AAG​CGT​AG
β-actin	CGT​TGA​CAT​CCG​TAA​AGA​CCT​C	TAG​GAG​CCA​GGG​CAG​TAA​TCT

### Statistical analysis

All experimental data were expressed as mean ± SEM. SPSS software v20.0 (IBM Corporation, Armonk, NY, United States) was utilized for statistical analyses, including one-way analysis of variance (ANOVA) and the least significant difference (LSD) *post hoc* test for multiple comparisons. The count data was analyzed by the nonparametric Kruskal-Wallis test and Mann-Whitney U *post hoc* test. It was deemed statistically significant when *p* < 0.05.

## Results

### MT suppressed spatial recognition and learning memory impairment in chronic sleep-deprived rats

The most obvious symptom of learning memory dysfunction is decreased spatial discrimination (Wang et al., 2019b) while the MWM test is one of the most commonly used tests to assess learning and memory abilities in rodents (He et al., 2021). Accordingly, we evaluated the spatial learning ability of all rats by the navigation test. After two training days, there was no significant difference in escape latency between animals in each group (*p* > 0.05, Figure 1C). Starting from day 3, a significant difference in escape latency between the control and model groups was observed (*p* < 0.05, Figure 1C). On day 4, the MT-H group showed a significant difference from the model group (*p* < 0.05, Figure 1C), while the MT-L group only showed a difference on day 5 (*p* < 0.01, Figure 1C). Figure 1D shows representative trajectories of the animals in each group, which demonstrated that MT significantly shortened the sleep deprivation-induced rats’ escape latency and that MT was superior to 50 mg/kg at a dose of 100 mg/kg. The platforms were removed on day 6 while the spatial probe test was used to evaluate the memory capacity of rats. The results showed that the time of crossing the platform was significantly greater in the MT-L and MT-H groups than in the model group (*p* < 0.05 or *p* < 0.001, Figure 1E). Meanwhile, the MT-L and MT-H groups significantly increased the time of rats in the target quadrant (*p* < 0.05 or *p* < 0.001, Figure 1F). SD-induced rats demonstrated irregular and chaotic movements in the spatial probe test, whereas the MT-treated group showed better memory ability (Figure 1D). These results revealed that SD impaired spatial recognition and learning memory in rats, and MT, especially MT-H, reversed this impairment.

### MT alleviated hippocampal nerve damage and neuron loss in chronic sleep-deprived rats

**FIGURE 2:**
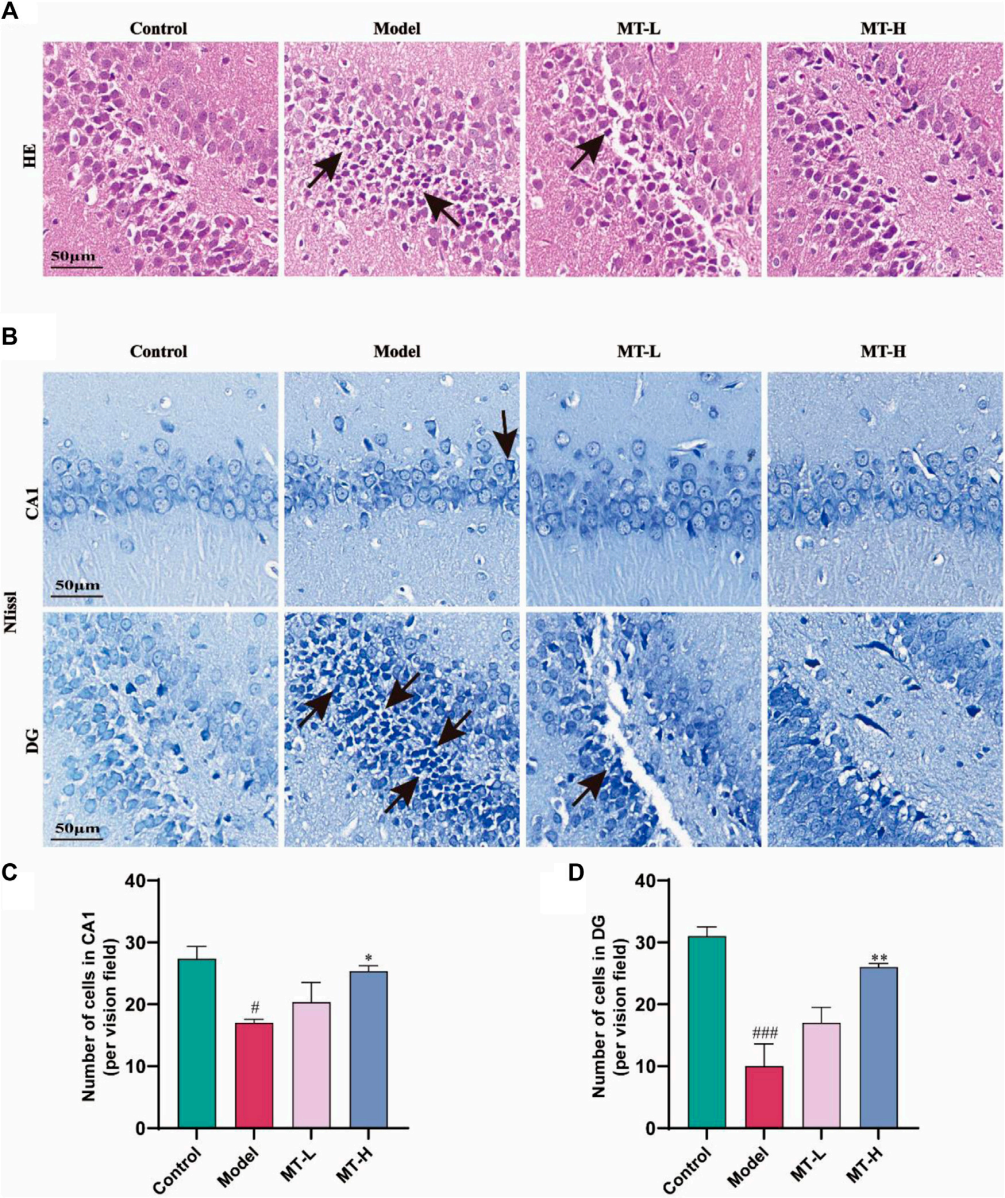
MT reversed hippocampal damage in sleep-deprived rats. **(A)** Representative images of H&E staining in the dentate gyrus (DG) region of HP. **(B)** Representative images of Nissl staining in the CA1 and DG region of HP. (**C, D)** Number of cells in CA1, and DG, respectively. The data are expressed as the means ± SEM. ^#^ *p* < 0.05, ^###^ *p* < 0.001 vs Control group; ^*^ *p* < 0.05, ^**^ *p* < 0.01 vs. Model group.

The MWM test has demonstrated that MT could suppress spatial recognition and learning memory impairment in chronic sleep-deprived rats. Therefore, HE staining and Nissl staining were used to assess hippocampal nerve damage in the animals. In HE staining, we found that hippocampal neurons in the control group were tightly arranged and no pathological damage was seen (Figure 2A), while the model group showed obvious pathological damage such as deepened staining and nuclear consolidation. Compared with the model group, the MT-L and MT-H groups had less neuronal injury and nuclear deformation. Nissl staining revealed that hippocampus neurons in the model group were sparsely arranged, there were fewer Nysted vesicles (Figure 2B), and the number of neurons in the CA1 and DG regions was lower than in the control group (*p* < 0.05 or *p* < 0.001, Figures 2C, D). However, The MT-L and MT-H groups showed less pathological damage than the model group (*p* < 0.05 or *p* < 0.01). The above results suggested that MT could alleviate hippocampus pathology and neuron loss in chronic sleep-deprived rats.

### MT inhibited microglia activation and Aβ42 aggregation to alleviate neuroinflammation in chronic sleep-deprived rats

**FIGURE 3:**
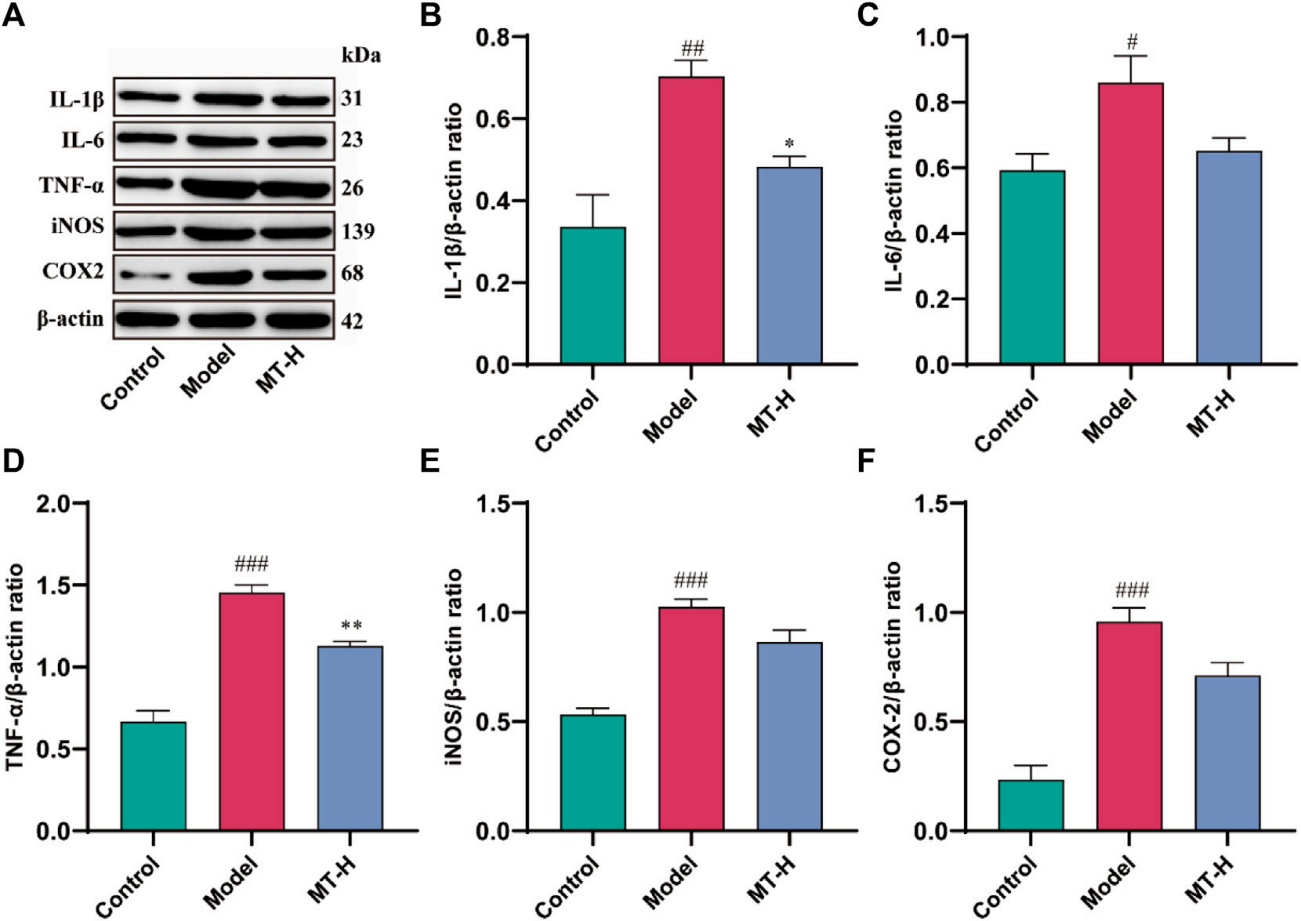
MT inhibited the production of pro-inflammatory proteins in sleep-deprived rats. **(A)** Western blot bands showing the protein expression levels of IL-1β, IL-6, TNF-α, iNOS, and COX2 in the HP, respectively. **(B–F)** Relative protein expression level of IL-1β, IL-6, TNF-α, iNOS, and COX2 in the HP, respectively. The data are expressed as the means ± SEM. ^#^ *p* < 0.05, ^##^ *p* < 0.01, ^###^ *p* < 0.001 vs Control group; ^*^ *p* < 0.05, ^**^ *p* < 0.01 vs. Model group.

**FIGURE 4:**
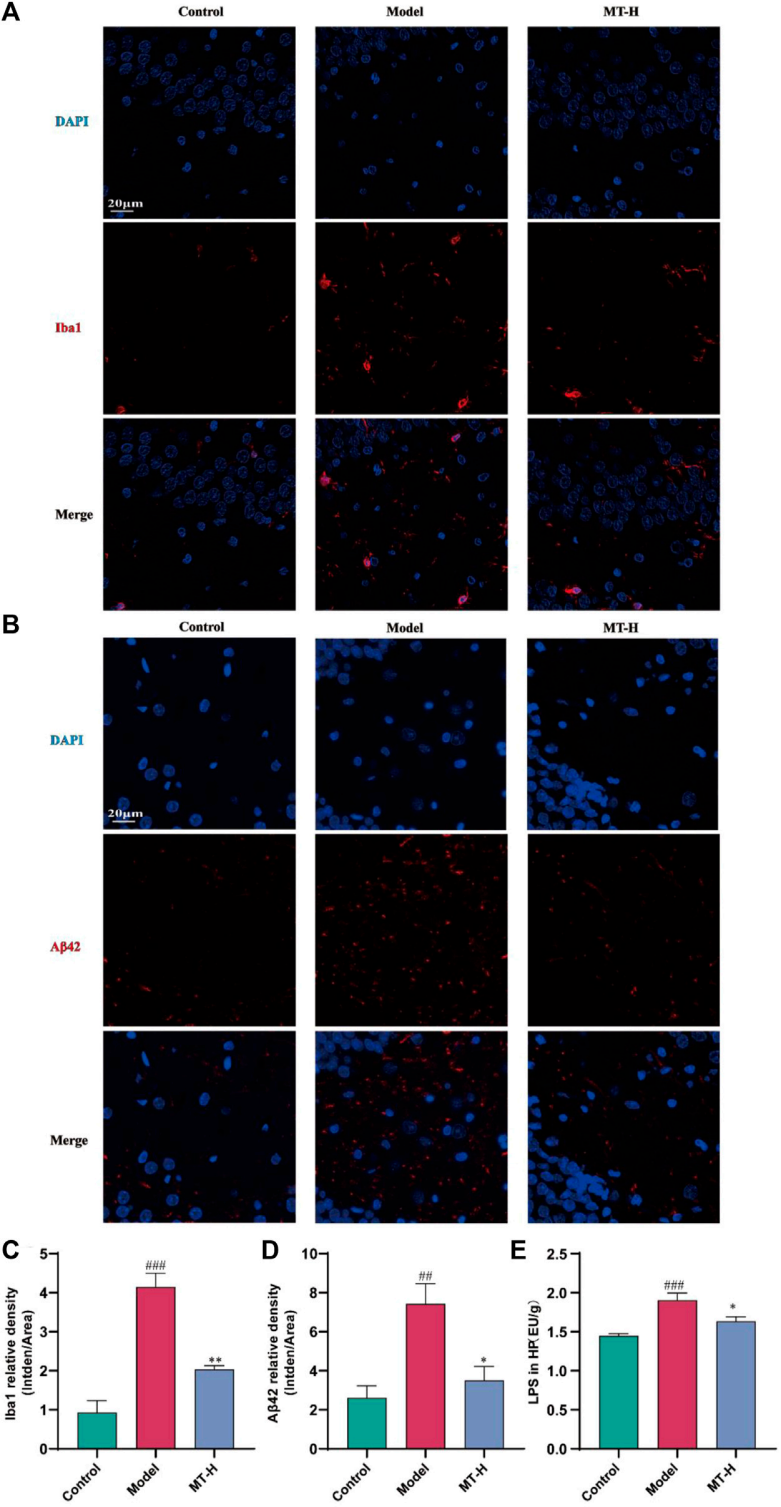
MT alleviated hippocampal Aβ42 deposition and microglia activation in sleep-deprived rats. **(A, B)** Representative immunofluorescence staining images of Iba1 and Aβ42 in the DG region of HP, respectively. **(C, D)** Relative density of Iba1 and Aβ42 in the DG region of HP, respectively. **(E)** LPS in HP. The data are expressed as the means ± SEM. ^##^ *p* < 0.01, ^###^ *p* < 0.001 vs. Control group; ^*^ *p* < 0.05, ^**^ *p* < 0.01 vs. Model group.

**FIGURE 5:**
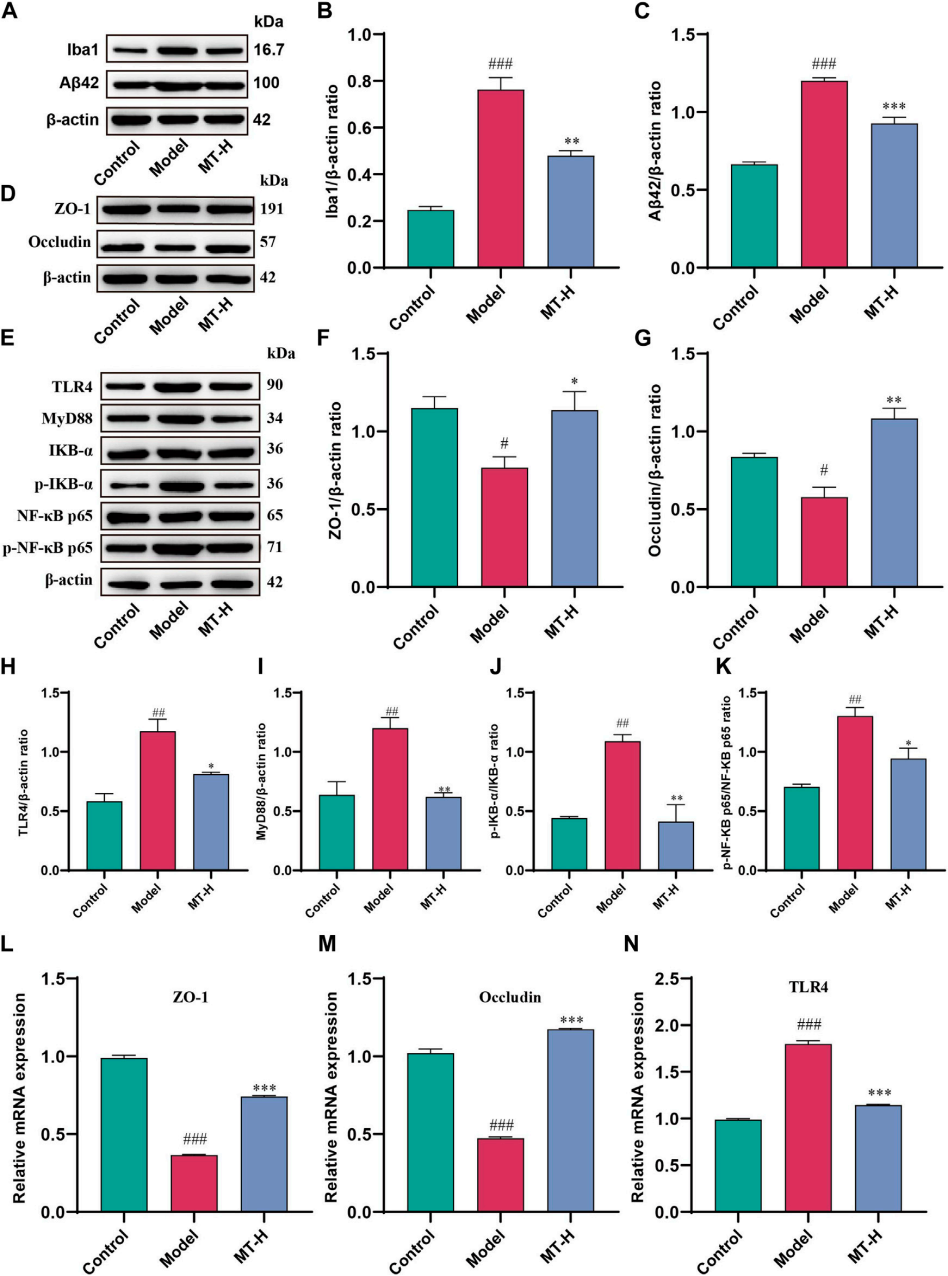
MT regulated brain barrier function and TLR4/MyD88/NF-κB signaling pathway in sleep-deprived rats. **(A)** Western blot bands showing the protein expression levels of Iba1 and Aβ42 in the HP, respectively. **(B, C)** Relative protein expression level of Iba1 and Aβ42 in the HP, respectively. **(D)** Western blot brands showing the protein expression levels of ZO-1 and occludin in the HP, respectively.**(E)** Western blot brands showing the protein expression levels of TLR4, MyD88, IKB-α, p-IKB-α, NF-κB p65, and p-NF-κB p65 in the HP, respectively. **(F, G)** Relative protein expression level of ZO-1 and occludin in the HP, respectively. **(H–K)** Relative protein expression level of TLR4, MyD88, p-IKB-α/IKB-α, and p-NF-κB p65/NF-κB p65, respectively. **(L–N)** Relative mRNA expression of ZO-1, occluding, and TLR4 in the HP, respectively. The data are expressed as the means ± SEM. ^#^ *p* < 0.05, ^##^ *p* < 0.01, ^###^ *p* < 0.001 vs. Control group;^*^ *p* < 0.05, ^**^ *p* < 0.01, ^***^ *p* < 0.001 vs. Model group.

Neuroinflammation contributes significantly to the appearance and progression of cognitive impairment (Wang et al., 2023). Therefore, we detected the levels of IL-1β, IL-6, TNF-α, iNOS, and COX2 in hippocampal tissues using Western blotting (Figure 3A). The model group showed higher levels of IL-1β, IL-6, TNF-α, iNOS, and COX2 compared with the control group (*p* < 0.05 or *p* < 0.01 or *p* < 0.001, Figures 3B–F), whereas these inflammatory and oxidative factors were suppressed after MT (100 mg/kg) treatment (*p* < 0.05 or *p* < 0.01). Microglial activation causes microglia to secrete high quantities of pro-inflammatory cytokines (Wang et al., 2023). According to the “Aβ cascade hypothesis,” the imbalance in Aβ synthesis and clearance can cause inflammation, oxidative stress, neurotoxicity, and neuronal apoptosis (Li et al., 2020). Therefore, we detected the levels of Iba1 and Aβ42 in hippocampal tissues by immunofluorescence (Figures 4A, B). The results showed that the fluorescence intensity of Iba1-positive microglia and Aβ42 was higher in the model group compared with the control group (*p* < 0.01 or *p* < 0.001, Figures 4C, D). MT treatment (100 mg/kg) drastically reduced Iba1-positive microglia and Aβ42 fluorescence intensity. Notably, we further verified the immunofluorescence results by Western blot (Figures 5A–C). Several studies have pointed out that elevated levels of LPS in the hippocampus are one of the key triggers of SD-induced neuroinflammation and cognitive impairment (Wang et al., 2023). Therefore, further ELISA experiments revealed high levels of LPS in the hippocampus of the model group compared to the control group (*p* < 0.001, Figure 4E). Furthermore, MT therapy (100 mg/kg) reduced hippocampal LPS levels (*p* < 0.05, Figure 4E). In summary, MT could inhibit microglia activation and Aβ42 aggregation to alleviate neuroinflammation in Chronic Sleep-deprived Rats.

### MT regulated brain barrier function and TLR4/MyD88/NF-κB signaling pathway in sleep-deprived rats

High levels of LPS and released inflammatory factors induced by SD disrupt the integrity of the blood brain barrier (BBB), accompanied by pathogenic factors that break through the BBB and enter the brain parenchyma (Wang et al., 2023). LPS entering the brain then bind to Toll-like receptor 4 (TLR4) receptors on microglia, initiating the downstream NF-κB signaling pathway via MyD88, leading to the secretion of large amounts of pro-inflammatory cytokines (Wang et al., 2023), resulting in more intense neuroinflammation and cognitive impairment. Therefore, we further investigated whether MT could protect the brain barrier function to alleviate neuroinflammation. Western blot revealed that SD rats had lower levels of ZO-1 and occludin in the hippocampus compared to the control group (*p* < 0.05 or *p* < 0.05, Figures 5D–G). However, MT treatment (100 mg/kg) reduced ZO-1 and occludin levels (*p* < 0.05 or ns). Further, qPCR demonstrated that mRNA expression of ZO-1 and occludin was decreased in the hippocampus of model rats compared to the control group (*p* < 0.001 or *p* < 0.001, Figures 5L, M), which was reversed by MT therapy (100 mg/kg) (*p* < 0.001 or *p* < 0.001). Previous studies have shown that activation of the NF-κB signaling pathway promotes neuroinflammation and neuronal apoptosis (Muhammad et al., 2019; Qu et al., 2021; Zhao et al., 2021), while inhibition of the pathway improves cognitive function (He et al., 2021). We therefore further investigated whether MT alleviates neuroinflammation by modulating the LPS-TLR4-mediated NF-κB signaling pathway. In this study, we firstly detected the levels of TLR4, MyD88, IKB-α, and NF-κB p65 in hippocampal tissues as well as the levels of p-IKB-α and p-NF-κB p65 by Western blot. The results showed that the levels of TLR4, MyD88, p-IKB-α, and p-NF-κB p65 were significantly elevated in the model group compared with the control group (*p* < 0.01 or *p* < 0.01 or *p* < 0.01 or *p* < 0.01, Figures 5H–K), indicating that the TLR4/MyD88/NF-κB signaling pathway was activated by SD, while MT treatment (100 mg/kg) inhibited the pathway (*p* < 0.05 or *p* < 0.01 or *p* < 0.01 or *p* < 0.05). Further qPCR showed that hippocampal TLR4 mRNA expression was reduced in model rats compared with the control group (*p* < 0.001, Figure 5N), whereas MT treatment (100 mg/kg) regressed the level of TLR4 mRNA (*p* < 0.001). The above results suggest that MT could protect brain barrier function and inhibit the activation of the TLR4/MyD88/NF-κB signaling pathway in sleep-deprived rats.

## Discussion

Cognitive ability and learning memory are affected by a variety of factors, including age (Yang X. et al., 2020), disease (Bi et al., 2021), diet (Sun et al., 2022), sleep (Wu et al., 2022), and genetics (Lindner et al., 2022). We have focused our attention on the issue of learning memory decline induced by sleep problems. Multiple studies have shown that cognitive and memory impairments are one of the most common symptoms of chronic sleep loss or disturbance (Wang et al., 2019a; Li et al., 2022). In this study, the 3-week modified multi-platform method was used to create the SD rat model (Sazak Kundi et al., 2021; Chen et al., 2022). The MWM test is generally used to assess long-term memory and spatial learning abilities, and rodents need to collect, process, and store spatially relevant cues to help escape from unpleasant water situations (Chen et al., 2017). Consistent with previous reports, various causes of SD or sleep disruption influenced animal behavior in WMW experiments, primarily in the form of prolonged escape latency during the navigation test (Zhang et al., 2022), as well as a decrease in the time of crossing the platform and time of crossing the platform during the spatial probe test (Zhang et al., 2022). Our findings revealed that different doses of MT therapy had a substantial effect on decreasing escape latency in a measure-dependent manner, resulting in a considerable increase in the number of crossing platforms and target quadrant time during the spatial probe test. Rats in the control and MT treatment groups (50 and 100 mg/kg) frequently gazed about the room or remained in position for a brief period before swimming to the platform, whereas rats in the model group exhibited blind and disordered swimming, as observed. Thus, MT not only alleviated SD-induced memory impairment, but also improved rats’ spatial learning abilities.

The maintenance of hippocampus-dependent learning memory capacity is intricately connected to the structure and function of neurons, and nerve damage may trigger cognitive impairment. To determine if MT may relieve SD-induced hippocampal neuropathological alterations, we detected neurons by HE staining and Nysted staining. In HE staining, we discovered that hippocampus neurons in SD model rats displayed deeper staining, malformed nuclei, and degenerative alterations. After 3 weeks of MT therapy, the pathological damage to the hippocampus region reversed. Combined with the results of ImageJ’s Nysted staining, the hippocampus neurons in the model group of rats were sparsely organized, with fewer neurons in the CA1 and DG areas than in the control group, suggesting that SD caused neuronal loss. The MT-L and MT-H treatments increased the number of neurons in the CA1 and DG areas compared to the model group, with only MT-H showing a significant difference (*p* < 0.05 or *p* < 0.01), The MT-L and MT-H treatments increased the number of neurons in the CA1 and DG areas compared to the model group, with only MT-H exhibiting a significant difference (*p* < 0.05 or *p* < 0.01), which prompted the selection of MT-H samples for subsequent biochemical experiments. Our findings indicated that MT inhibited SD-induced neurodegenerative alterations and neuronal death in the hippocampus in a dose-dependent way.

Neuroinflammation contributes significantly to deteriorate learning and memory abilities in SD (Jin et al., 2019; Li et al., 2022). Established reports have emphasized the importance of microglia in forming memories and supporting neurogenesis in the brain. Early in the disease process, microglia are able to phagocytose and clear accumulated Aβ. However, with the persistence of chronic sleep deprivation and the establishment of a chronic inflammatory state, the M1 (pro-inflammatory) or M2 (antiinflammatory) state of microglia is abnormally activated, accompanied by the continuous production of large amounts of pro-inflammatory cytokines (Jin et al., 2019), and eventually resulting ineffective phagocytosis of Aβ(Milligan Armstrong et al., 2021). The imbalance between Aβ production and clearance causes Aβ deposition, leading to neuroinflammation, neurotoxicity, and neuronal loss (Yang J. et al., 2020; Li et al., 2020). Activated microglia-induced Aβ deposition and neurofibrillary tangles indirectly drive cognitive decline. Our study found that MT reduced inflammatory cytokine production in the hippocampus produced by SD, including IL-1β, IL-6, TNF-α, iNOS, and COX2. Immunofluorescence and Western blot analysis revealed that MT inhibited hippocampus microglia activation, as well as Aβ42 expression and deposition. Therefore, we concluded that neuroinflammation was implicated in the process of SD-induced hippocampal lesions. MT could successfully prevent SD-induced microglia activation and Aβ42 deposition, as well as heal hippocampus inflammatory damage.

Several investigations have revealed that LPS is causally associated with neuroinflammation and neurodegenerative disorders in rodents (Yang J. et al., 2020). LPS has been connected with pro-inflammatory factors and alteration of brain barrier function in neuroinflammation (Qu et al., 2021). In the brain, TLR4 is widely expressed on the surface of microglia and is involved in the recognition of LPS, which is mediated by the adapter MyD88 (Yang J. et al., 2020). This facilitates the entry of cytoplasmic NF-κB into the nucleus and promotes the activation of the NF-κB complex, which then involves the transcriptional expression of downstream inflammatory cytokines. TLR4-initiated signaling mediates the phosphorylation and degradation of IKB-α, which allows the NF-κB p65 subunit to shuttle into the nucleus, where it binds to specific DNA shared sequences, thereby enhancing the transcription of inflammation-associated proteins (Jin et al., 2019), and LPS is a key inducer of this signaling pathway. Brain dysfunction can activate APP metabolic pathways, including activation of β and γ secretases to promote Aβ production, resulting in further deterioration of cognitive dysfunction (Kempuraj et al., 2019). In addition, stressors such as SD have been reported to cause neurovascular damage and increase the permeability of the BBB(Serna-Rodriguez et al., 2022; Puech et al., 2023). Impaired barrier function allows for wider diffusion of LPS and pro-inflammatory factors in the brain, triggering a vicious cycle that leads to more severe inflammatory brain damage. Our results demonstrated that MT reversed SD-induced elevated levels of LPS and barrier function impairment in the hippocampus while upregulating the expression of barrier proteins ZO-1 and occludin. Further, Western blot was utilized to assess if MT impacts TLR4/NF-κB signaling. We found that TLR4 was activated in SD rats, as indicated by its upregulation, contributing to the downstream activation of NF-κB signaling, characterized by enhanced phosphorylation of IκB-α and NF-κB p65 expression. In contrast, MT significantly inhibited the upregulated expression of TLR4, the phosphorylation and degradation of IκB-α, and the subsequent nuclear translocation of NF-κB p65. In addition to the protein level, MT also strongly inhibited the upregulated expression of TLR4 mRNA. Based on these results, we conclude that MT intervention in SD-induced neuroinflammation and cognitive dysfunction is mediated through the downregulation of the TLR4/MyD88/NF-κB signaling pathway.

## Conclusion

**FIGURE 6:**
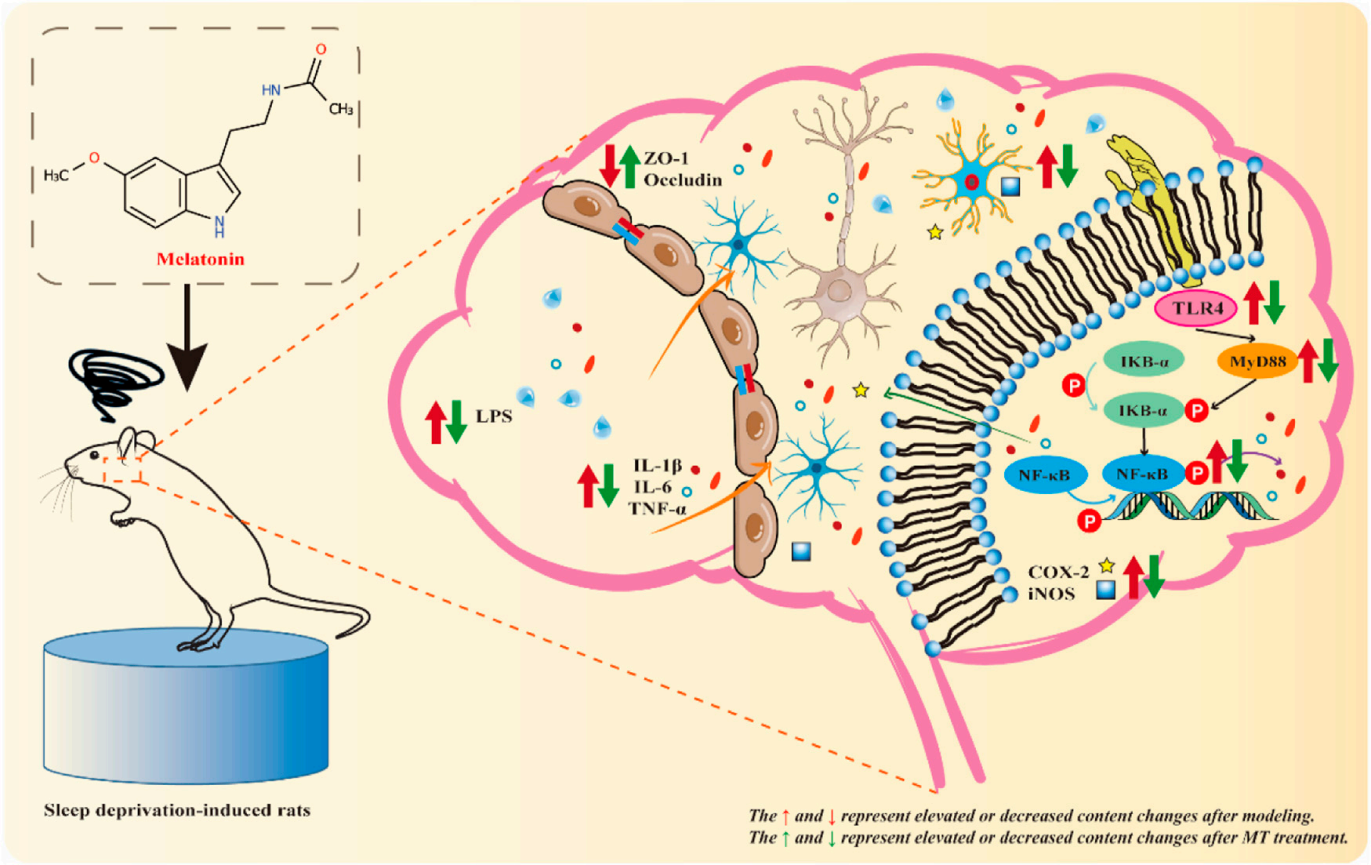
Mechanistic diagram. Sleep deprivation activates the hippocampal TLR4/MyD88/NF-κB signaling pathway in rats, resulting in hippocampal inflammatory damage and cognitive dysfunction. MT may exert neuroprotective effects by down-regulating the levels of LPS and Aβ42, up-regulating the expression of ZO-1 and Occludin, inhibiting the activation of microglia and the TLR4/MyD88/NF-κB signaling pathway, and reducing the release of pro-inflammatory indicators (IL-1 β, IL-6, TNF-α, iNOS, and COX2) to exert neuroprotective effects and improve chronic sleep deprivation-induced neuroinflammation and cognitive dysfunction.

Based on the above series of results, we suggest that MT may exert neuroprotective effects by inhibiting the TLR4/MyD88/NF-κB signaling pathway to ameliorate chronic sleep deprivation-induced neuroinflammation and cognitive dysfunction. Thus, MT may offer a promising therapeutic strategy in the prevention of sleep deprivation-related cognitive impairment (Figure 6).

## Data Availability

The original contributions presented in the study are included in the article/Supplementary Material, further inquiries can be directed to the corresponding authors.

## Abbreviations

BBB, blood brain barrier; CMC-Na, Carboxymethyl Cellulose Sodium; DG, dentate gyrus; ELISA, Enzyme-linked immunosorbent assay; HBUCM, Hubei University of Chinese Medicine; H&E, hematoxylin and eosin; HP, hippocampus; LPS, Lipopolysaccharide; MT, Melatonin; TLR4, Toll-like receptor 4; qPCR, Quantitative real-time polymerase chain reaction; SD, Sleep deprivation; ZO-1, Zonula occluden 1.
